# Interleukin-6 moderates the relationship between social support, strain, and future depressive symptoms

**DOI:** 10.1016/j.bbih.2025.101122

**Published:** 2025-10-13

**Authors:** Timothy Bang Hao Aw, Nur Hani Zainal

**Affiliations:** National University of Singapore, Department of Psychology, Singapore

**Keywords:** Depression, Inflammation, Social support, Strain, Moderator

## Abstract

**Background:**

Inflammation has been increasingly implicated in major depressive disorder (MDD), with interleukin-6 (IL-6) emerging as a key biomarker. How this relates to psychosocial risk factors, such as social support and strain, remains underexplored. IL-6 levels, social support, and strain may interact through shared underlying mechanisms in conferring depression risk. The study examined whether IL-6 levels moderate the associations between social support and strain with future MDD symptoms.

**Methods:**

Longitudinal data from 1,054 community adults in the Midlife Development in the United States (MIDUS) study were analyzed. Multiple linear regression models examined the main and interactive effects of social support, social strain, and IL-6 on future MDD symptoms. Serum IL-6 levels were measured using both enzyme-linked immunosorbent assay (ELISA) and Meso Scale Discovery (MSD) immunoassays, with results cross-examined. Sensitivity analyses, including generalized additive models (GAM) and covariate-adjusted models, were conducted to account for potential nonlinearities and confounders.

**Results:**

Lower social support and greater social strain predicted higher future MDD symptoms, particularly among individuals with elevated IL-6 levels. The interaction between social strain and IL-6 levels was robust across both ELISA (*d* = 0.18, *p* = .003) and MSD-derived (*d* = 0.12, *p* = .048) assay methods. In contrast, the interaction between social support and IL-6 was observed only in ELISA-based measurements (*d* = −0.13, *p* = .033).

**Conclusion:**

IL-6 levels may moderate the relationship between social support, strain, and future MDD symptoms. Interpersonally-focused interventions enhancing social support or mitigating strain may consider the potential interacting role of inflammation in alleviating the psychosocial risk of depression.

## Highlights

• IL-6 increases the 9-year social strain-to-depression link across assay methods.
• Social support mitigates MDD severity risk at medium-to-high IL-6 levels.
• The 9-year design detects interactions between inflammation and social strain.
• MSD assays show independent impacts of both social strain and support.
• IL-6 may moderate the risk of MDD via the HPA axis and neuroimmune pathways.

## 1 Interleukin-6 Moderates the Relationship between Social Support, Strain, and Future Depressive Symptoms

Major depressive disorder (MDD) is a prevalent psychiatric condition and is the second leading contributor to the global burden of disease (Vos et al., 2012). It is a key risk factor for suicide (Dong et al., 2019) and the leading cause of years lived with disability (Friedrich, 2017; Otte et al., 2016). Individuals with MDD typically exhibit symptoms such as persistent low mood, anhedonia, appetite changes, and sleep disturbances (Gigantesco and Morosini, 2008). These symptoms also appear in various subclinical syndromes in the general population (Biella et al., 2019; Fried, 2015). Although less severe, these subclinical syndromes represent part of a continuum of depression severity that leads to worse health outcomes as symptoms accrue (Ayuso-Mateos et al., 2010). This pattern suggests that key factors contributing to the disease burden of depression, such as reduced productivity, social dysfunction, and functional impairment (Chow et al., 2022; Lepine and Briley, 2011), arise at different levels of symptom severity in the general population. Therefore, identifying risk factors for increased MDD symptom severity in the general population is crucial for efforts aimed at mitigating the total disease burden of depression.

Inflammation is an established risk factor for MDD and plays a significant role in the etiology and pathophysiology of MDD symptoms (Lee and Giuliani, 2019; Wohleb et al., 2016). When acute, the proinflammatory process is brief and subsides once the threat has passed (Ahmed, 2011). However, frequent and prolonged initiation of the proinflammatory response hinders immune resolution and may lead to the dysregulation of key physiological processes (Leonard, 2018; Troubat et al., 2021). A key marker of inflammation in depression is interleukin-6 (IL-6), a proinflammatory cytokine involved in the transition from acute to chronic low-grade inflammation (Roohi et al., 2021; Schett, 2018). According to the cytokine theory (Dantzer et al., 2008), proinflammatory cytokines such as IL-6 may alter brain activity in ways that induce MDD symptoms over time. For instance, the overexpression of IL-6 may contribute to the development of cognitive-affective symptoms by reducing serotonin synthesis (Troubat et al., 2021) and increasing serotonergic turnover (Kopschina Feltes et al., 2017). Moreover, elevated IL-6 has been shown to disrupt hypothalamic-pituitary-adrenal (HPA) axis regulation and affect levels of cortisol secretion (Cheiran Pereira et al., 2022; Menke, 2024), inducing somatic symptoms such as appetite changes and disrupted sleep (Chu et al., 2019; Jokela et al., 2016). Lastly, peripheral IL-6 levels may reflect neural patterns associated with specific depressive phenotypes. For instance, higher levels of IL-6 are associated with increased connectivity within the default mode network (Marsland et al., 2017a), which has been linked to excessive rumination and self-referential cognitions in depression (Zeng et al., 2023).

Meta-analytic findings of both cross-sectional (Dowlati et al., 2010; Goldsmith et al., 2016; Howren et al., 2009; Kohler et al., 2017; Osimo et al., 2020; Smith et al., 2018; Strawbridge et al., 2015) and longitudinal studies (Mac Giollabhui et al., 2021; Valkanova et al., 2013) consistently show that higher inflammation levels are concurrently and prospectively associated with higher levels of MDD symptoms. These findings are most consistently observed in markers of IL-6 (Dowlati et al., 2010; Mac Giollabhui et al., 2021), which may be attributed to its role in recruiting most acute-phase proteins (Gabay, 2006) and in mediating the chronic proinflammatory response via immune cell recruitment (Kaplanski et al., 2003). This suggests that levels of IL-6 may be prospectively associated with future MDD symptoms.

However, cytokine activity and signaling changes are implicated in nearly all factors that predispose individuals to or trigger depression (Himmerich et al., 2019; Ting et al., 2020). Moreover, risk factors often converge on shared underlying mechanisms in conferring depression risk (Cui et al., 2024; Funkhouser et al., 2021). This suggests potential interactions between risk factors that maintain otherwise independent associations (Lasselin, 2021). For instance, recent evidence has shown that the depressogenic effect of inflammation may interact with psychosocial factors, such as early-life stress (Kuhlman et al., 2020), childhood adversity (Zainal and Newman, 2021), and trait sensitivity to social disconnection (Irwin et al., 2019).

Central to this study are the potential interacting roles of social support and strain. Social support is a key protective factor against depression and maintains an independent association with MDD symptom risk (Brown et al., 2012; Hughes et al., 2014). According to the buffering hypothesis (Cohen and Wills, 1985), social support confers resilience to psychosocial stressors by buffering the physiological effects of stress reactions (Hostinar et al., 2014). For instance, social support may downregulate HPA axis activation by stimulating the release of oxytocin, which reduces cortisol secretion (Heinrichs et al., 2003; Rosal et al., 2004). Additionally, social support is known to minimize autonomic activation and modulate monoamine activity in response to stress (Ditzen and Heinrichs, 2014). These findings suggest that social support may attenuate physiological processes that mediate the inflammation-depression pathway, a relationship consistent with moderation (Kraemer et al., 2001). Relatedly, higher social strain is a psychosocial factor that may be associated with more inflammation-related MDD symptoms over time (Shin and Gyeong, 2023). Although lower social support might represent the absence of a buffering effect, higher social strain may reflect levels of chronic stress exposure that directly exacerbate proinflammatory cytokine activity over time (Yang et al., 2014). This is supported by recent studies examining social support and strain, which suggest that they independently contribute to the development and severity of MDD symptoms (Lerman Ginzburg et al., 2021; Mussa et al., 2024).

The present study builds on these findings by examining the relative contribution of inflammation, social support, and social strain in predicting nine-year MDD symptoms. Previous studies have typically examined these factors independently or employed cross-sectional designs, limiting causal inference (Pearl, 2014). We also extend prior research on the potential interacting roles of social support, strain, and inflammation in shaping vulnerability to MDD symptoms within a broad adult population. Given existing theories, research, and knowledge gaps, the present study's hypotheses were threefold. First, we hypothesized that lower social support, higher social strain, and higher levels of inflammation would each be independently associated with greater future MDD symptoms (main effects; H_1_). Second, we hypothesized that the relationship between social support and future MDD symptoms would be moderated by the level of inflammation, such that higher inflammation would strengthen the relationship between lower social support and future MDD symptoms (interaction effect 1; H_2_). Third, we anticipated that the relationship between social strain and future MDD symptoms would be moderated by the level of inflammation, such that higher inflammation would amplify the relationship between higher social strain and future MDD symptoms (interaction effect 2; H_3_).

## 2 Methods

### 2.1 Participants

Participants comprised 1,054 community-dwelling adults who participated in the Midlife Development in the United States (MIDUS) study, which included two assessment waves that were relevant to the current secondary analysis (Ryff et al., 2019; Weinstein et al., 2019). At baseline, participants had a mean age of 55.19 ± 11.81 years, ranging from 25 to 74. The sample comprised 477 (45.3%) males and 577 (54.7%) females. Regarding education, 465 (44.1%) of the sample had completed a college or university degree, 300 (28.5%) had completed some college, 238 (22.6%) had attained a high school education, and the remaining 51 (4.8%) had either not completed high school or did not disclose their level of education.

### 2.2 Procedure

All participants completed both waves of the MIDUS II Biomarker Study (Love et al., 2010; Ryff et al., 2019). MDD symptoms were assessed via clinical interviews at Wave 1 (W1; 2004 to 2009) and Wave 2 (W2; 2013 to 2014). Social support, strain, and other demographic variables were assessed via a self-administered questionnaire at W1. IL-6 levels were measured as part of a two-day biomarker protocol at one of three General Clinical Research Centers.

### 2.3 Measures

**W1 and W2 MDD symptoms.** MDD symptoms were assessed through the World Health Organization (WHO) Composite International Diagnostic Interview Short Form (CIDI-SF; Kessler et al., 2006). MDD symptoms were based on the criteria outlined in the third revised edition of the Diagnostic and Statistical Manual of Mental Disorders (DSM-III-R; Spitzer et al., 1992). CIDI-SF scores reflected the presence or absence of specific MDD symptoms, including anhedonia, changes in appetite, fatigue, difficulty concentrating, feelings of self-criticism, sleep disturbances, and suicidal thoughts in the past 12 months. Scores ranged from 0 (*no reported symptoms*) to 7 (*presence of all symptoms assessed*). The scale demonstrated high internal consistency (α = .930 herein) and has evidenced strong construct validity (Kessler et al., 2006).

**W1 Social** **support.** Participants assessed the extent to which they had received support from their spouse or partner (if applicable), family members, and friends. They rated how much each source had provided support by offering care, understanding their feelings, being dependable, and the extent to which participants felt they could open up to them. The Spouse or Partner Support Scale included two additional items: whether their spouse/partner had appreciated them and whether participants felt they could be themselves around them. Each item was rated on a four-point scale, from 1 (*not at all*) to 4 (*a lot*). 10.13039/501100007185Total social support was calculated by combining the three support scales, yielding a theoretical range of 14–56. The scale demonstrated strong internal consistency (α = .787 herein) and has good discriminant validity (Creaven et al., 2020).

**W1 Social strain.** Participants assessed the extent to which they felt that their spouse or partner, family members, and friends had made excessive demands, criticized them, failed to meet their expectations, or caused them irritation. The Spouse or Partner Strain Scale included two additional items: whether their spouse/partner argues with them and whether they make them feel tense. Each item was rated on a four-point scale, from 1 (*not at all*) to 4 (*a lot*). Total strain was calculated by combining the three scales of strain, yielding a theoretical range of 14–56. The scale showed strong internal consistency (α = .776 herein) and has good convergent and discriminant validity (Fitzgerald and Morgan, 2022; Teo et al., 2013).

**W1 Serum IL-6.** Venous blood samples were obtained by venipuncture by a certified phlebotomist into 10 mL Becton Dickinson (BD) vacutainers (#VS367839) following an overnight fast. The samples were centrifuged and stored in a freezer maintained at −60 °C to −80 °C at one of three General Clinical Research Centers: the University of California, Los Angeles (UCLA), the University of Wisconsin, and Georgetown University. Samples were then shipped to the MIDUS BioCore Laboratory (University of Wisconsin, Madison, WI), where they were stored at −65 °C until assayed. Serum IL-6 concentrations were principally assayed using the Quantikine® High-Sensitivity enzyme-linked immunosorbent assay (ELISA) (#HS600B). Absorbance was measured at 490 nm using a Dynex Technologies Measurement of Relative Absorbance (MRX) II Microplate Reader (#1CXD4268). In consideration of variable precision, sensitivity, and reproducibility of IL-6 measurements across immunoassay platforms (Lasseter et al., 2020), we also included newly added serum IL-6 concentrations in the MIDUS dataset measured by electrochemiluminescence (Hartanto et al., 2021). Serum IL-6 concentrations in the MIDUS dataset were thus additionally assayed using the V-Plex Custom Cytokine Kit (#K151A0H-2) (Meso Scale Discovery [MSD], Rockville, MD), with a 96-well multispot plate and Mass Spectrometry Detection Sector Imager (#HTS24). Values exceeding the upper limit of quantification (ULOQ) were replaced with the corresponding ULOQ value for each assay (ELISA: 23 pg/mL; MSD: 145.05 pg/mL) (Lasseter et al., 2020). These values were then log-transformed to correct for deviations from normality, with all subsequent analyses conducted on the transformed values. Intra-assay and inter-assay coefficients of variation were within acceptable limits for both ELISA-derived IL-6 measurements (Friedman et al., 2005) and MSD-derived methods.

### 2.4 Data analysis

All analyses were performed using *RStudio* software (R Core Team, 2024). Missing data (7.4%) were handled through multiple imputation under the assumption that the data were missing at random (Lee and Shi, 2021). Regression and moderator diagnostics, including tests for multivariate normality, linearity of predictors, homoscedasticity, and independent residual variances (Hainmueller et al., 2018; Karazsia et al., 2014), were conducted prior to the analysis. These preprocessing steps and assumption checks suggested that the data were suitable for the present research aims.

For our preliminary analyses, we examined cross-sectional relationships among IL-6 levels, social support, and social strain at W1 using Pearson product-moment correlations. We then evaluated within-subject changes in MDD symptoms, IL-6 concentrations, social support, and social strain between W1 and W2 through paired-samples *t*-tests. Although IL-6 data were collected in both assessment waves, our primary analyses focused on how W1 IL-6 levels predicted MDD severity at W2, in line with our central hypotheses. To complement these analyses, we assessed whether mean IL-6 levels differed significantly between W1 and W2.

To test our hypotheses, multiple linear regression models were performed, including four predictor terms (IL-6, social support, social strain, and W1 MDD symptoms) and two interaction terms (IL-6 × social support and IL-6 × social strain) to examine the relationship between these variables and W2 MDD symptoms. Our first hypothesis was evaluated by examining the main effects of social support, social strain, and inflammation on predicting future MDD symptoms (H_1_) across both ELISA and 10.13039/100030732MSD IL-6 concentrations. Our second and third hypotheses, which tested relationships consistent with moderation, were assessed by the significance of the interaction terms in the regression model and by subsequent moderator analyses (H_2_ and H_3_) (Aguinis and Gottfredson, 2010; Yuan et al., 2014).

Sensitivity analyses using generalized additive models (GAM) with the *mgcv* package (Wood, 2017) were performed to examine potential non-linear relationships between variables. The *s*() smooth function was applied to model interaction terms, allowing for the detection of non-linear interaction effects on future MDD symptoms. Smoothing parameters were estimated via the restricted maximum likelihood estimator (REML) based on model residuals (Maestrini et al., 2024; Wood, 2011). Model diagnostics and visual plots were subsequently generated to illustrate significant non-linear associations. To aid interpretation, standardized effect sizes were derived by calculating Cohen's *d* using the formula *d* = 2*t*/√(*df*), where *t* represents the test statistic for the specific parameter estimate, and *df* denotes the model's degrees of freedom (Dunst et al., 2004; Rosenthal, 1994). To account for multiple comparisons, we applied a Bonferroni correction procedure (Simes, 1986).

## 3 Results

### 3.1 Initial analyses

**Zero-order correlations.** Demographic, clinical, biological, and psychosocial characteristics of the sample across both waves are described in Table 1. Table S1 in the online supplemental materials (OSM) shows the zero-order correlations among key variables at W1. Both ELISA and MSD IL-6 were positively associated at W1 (*r* = 0.58, *p* < .001). ELISA IL-6 was modestly, though positively, associated with both social support (*r* = 0.09, *p =* .005) and social strain (*r* = 0.09, *p* = .004). In contrast, 10.13039/100030732MSD IL-6 was not significantly associated with either social support (*r* = 0.03, *p =* .309) or social strain (*r* = 0.05, *p* = .110). Finally, both social support and social strain were positively associated (*r* = 0.74, *p* < .001) at W1.

**Table 1:** Participant demographic, clinical, biological, and psychosocial characteristics (*N* = 1054).

Variables	Wave 1 (W1)	Wave 2 (W2)
Demographics		
Age (in years)^a^	55.19 ± 11.81	64.19 ± 11.81
Gender (male)^b^	477 (45.26%)	477 (45.26%)
Ethnicity (White/non-Hispanic)^b^	961 (91.18%)	961 (91.18%)
Education (at least high school)^b^	1003 (95.16%)	1003 (95.16%)
Clinical		
MDD symptoms^a^	0.62 ± 1.87	0.51 ± 1.72
Body mass index^a^	27.95 ± 5.60	28.20 ± 6.04
Presence of smoking history^b^	471 (44.69%)	450 (42.70%)
Number of chronic conditions^a^	2.30 ± 2.34	3.31 ± 3.11
Medication use^a^	1.42 ± 1.50	1.43 ± 1.49
Medical treatment visits^a^	3.66 ± 4.05	4.22 ± 8.08
Mental health treatment visits^a^	2.18 ± 8.87	1.49 ± 6.37
Biological		
ELISA IL-6 (pg/mL)^a^^,^^c^	0.70 ± 0.65	1.44 ± 0.54
MSD IL-6 (pg/mL)^a^^,^^c^	0.17 ± 0.56	0.86 ± 0.54
Psychosocial		
Social support^a^	46.86 ± 10.08	48.85 ± 10.76
Social strain^a^	30.51 ± 12.73	32.17 ± 14.10

*Note*. MDD = major depressive disorder; ELISA = enzyme-linked immunosorbent assay; IL-6 = interleukin-6; MSD = Meso Scale Discovery.

a Mean ± standard deviation.

b Frequency (percentage).

c Log-transformed values.

**Differences between W1 and W2.** Between W1 (*M* = 0.62, *SD* = 1.87) and W2 (*M* = 0.51, *SD* = 1.72), there was no significant change in MDD symptoms, mean difference = 0.11, 95% CI [–0.01, 0.24], *d* = 0.05, *p* = .078. Conversely, social support increased significantly between W1 (*M* = 46.86, *SD* = 10.08) and W2 (*M* = 48.85, *SD* = 10.76), mean difference = 1.99, 95% CI [1.43, 2.55], *d* = 0.19, *p* < .001. Similarly, social strain increased significantly from W1 (*M* = 30.51, *SD* = 12.73) to W2 (*M* = 32.17, *SD* = 14.10), mean difference = 1.66, 95% CI [1.02, 2.29], *d* = 0.12, *p* < .001. Finally, IL-6 levels significantly increased across both assays: ELISA-based IL-6 levels increased from W1 (*M* = 0.70, *SD* = 0.65) to W2 (*M* = 1.44, *SD* = 0.54), mean difference = 0.75, 95% CI [0.71, 0.79], *d* = 1.24, *p* < .001; and MSD-based IL-6 levels increased from W1 (*M* = 0.17, *SD* = 0.56) to W2 (*M* = 0.86, *SD* = 0.54), mean difference = 0.68, 95% CI [0.64, 0.71], *d* = 1.27, *p* < .001.

### 3.2 Evaluation of study hypotheses

**IL-6 quantification via ELISA.** The multiple linear regression model, which included four predictor terms (IL-6, social support, social strain, and W1 MDD symptoms) and two interaction terms (IL-6 × social support and IL-6 × social strain) accounted for 13.6% of the variance in future MDD symptoms, *F*(6, 1047) = 28.65, *p* < .001 (see Table 2). Depressive symptoms at W1 were the strongest predictor of W2 MDD symptoms (*d* = 0.62, *p* < .001). The main effects of IL-6 (*d* = 0.08, *p* = .194), social support (*d* = −0.07, *p* = .246), and social strain (*d* = 0.05, *p* = .414) did not significantly contribute to future MDD symptoms (tests of H_1_). However, these null main effects were qualified by significant interactions between IL-6 × social strain (*d* = 0.18, *p* = .003; tests of H_2_) and IL-6 × social support (*d* = −0.13, *p* = .033; tests of H_3_).

**Table 2:** Multiple regression model of W1 ELISA IL-6 levels, social support, and strain on W2 MDD symptoms.

**Linear model estimates**					
Parametric coefficients	*b*	*(SE)*	*t*	*p*	*d*

(Intercept)	0.593	(0.374)	1.586	.113	0.098
W1 MDD Symptoms	0.273∗∗∗	(0.027)	10.032	<.001	0.620
W1 Social support	−0.013	(0.011)	−1.161	.246	−0.071
W1 Social strain	0.007	(0.009)	0.818	.414	0.051
W1 IL-6	0.486	(0.374)	1.300	.194	0.080
W1 Social support × IL-6	−0.023∗	(0.011)	−2.139	.033	−0.132
W1 Social strain × IL-6	0.025∗∗	(0.009)	2.927	.003	0.181

$R^{2}$	0.141				
Adjusted $R^{2}$	0.136				
*F*-statistic	28.65∗∗∗				
*p*	< .001				

**Generalized additive model (GAM) non-linear estimates**					
Parametric coefficients	*b*	*(SE)*	*t*	*p*	*d*

(Intercept)	–	(−)	–	–	–
W1 MDD Symptoms	0.272∗∗∗	(0.027)	10.098	< .001	0.624
W1 Social support	−0.050∗∗	(0.017)	−3.030	.003	−0.187
W1 Social strain	0.089∗∗∗	(0.026)	3.466	< .001	0.214
W1 IL-6	–	(−)	–	–	–

Significance of smooth terms	*edf*	*rdf*	*F*	*p*	

s(W1 Social support × IL-6)	8.637	11.376	1.725	.056	
s(W1 Social strain × IL-6)	6.216	8.859	2.795∗∗	.003	

Rank	59/62				
Adjusted $R^{2}$	0.171				
Deviance explained	18.40%				

*Note*. ∗*p* < .05, ∗∗*p* < .01, ∗∗∗*p* < .001.

W1 = wave 1; ELISA = enzyme-linked immunosorbent assay; IL-6 = interleukin-6; W2 = wave 2; MDD = major depressive disorder; *edf* = estimated degrees of freedom; *rdf* = reference degrees of freedom; *F* = *F*-ratio statistic.

s() = smoothed terms to accommodate any non-linear relations.

**Fig. 1:**
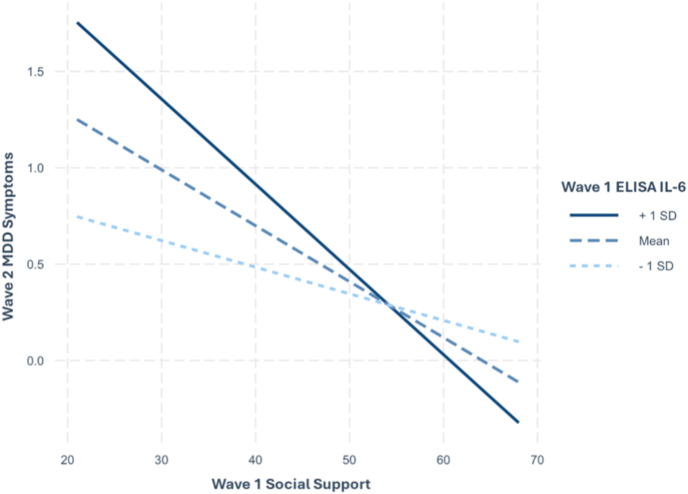
Simple Slopes of W1 Social Support predicting W2 MDD Symptoms at Levels of ELISA IL-6 *Note*. W1 = wave 1; W2 = wave 2; MDD = Major depressive disorder; ELISA = enzyme-linked immunosorbent assay; IL-6 = interleukin-6.

**Fig. 2:**
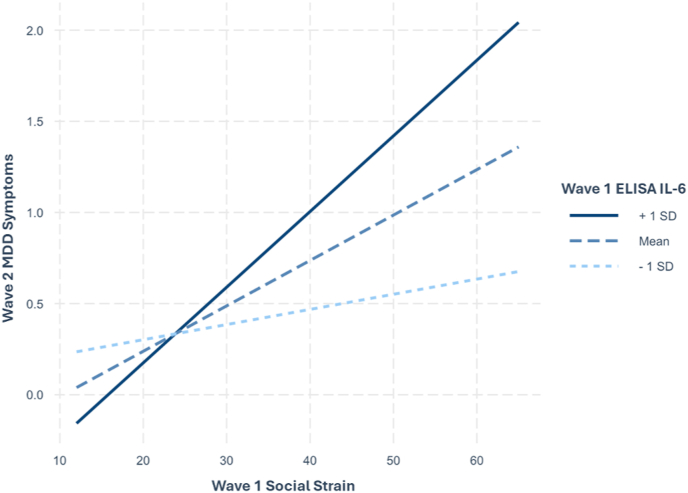
Simple Slopes of W1 Social Strain predicting W2 MDD Symptoms at Levels of ELISA IL-6 *Note*. W1 = wave 1; W2 = wave 2; MDD = Major depressive disorder; ELISA = enzyme-linked immunosorbent assay; IL-6 = interleukin-6.

Simple slope analyses were conducted to examine the interaction between IL-6 and social support, and between IL-6 and social strain. The simple slopes were calculated at the mean and ±1 standard deviation values of IL-6. In the present sample, levels of log-transformed IL-6 concentrations were categorized as low (*x* ≤ 0.044), medium (0.044 < *x* < 1.347), and high (1.347 ≤ *x*). Social support predicted future MDD symptoms at high (*d* = −0.28, *p* < .001) and medium (*d* = −0.24, *p* < .001) levels of IL-6 but not at low levels (*d* = −0.08, *p* = .200; Fig. 1). Similarly, social strain predicted future MDD symptoms at high (*d* = 0.530, *p* < .001) and medium (*d* = 0.26, *p* < .001) levels of IL-6 but not at low levels (*d* = 0.06, *p* = .330; Fig. 2). GAMs partially corroborate these findings when examining non-linear associations, with the interaction of IL-6 × social strain remaining significant and IL-6 × social support becoming nonsignificant (Table 2 and Fig. S1).

**IL-6 quantification via** 10.13039/100030732**MSD****.** Partial support for the study hypotheses was observed in IL-6 levels quantified through 10.13039/100030732MSD. The multiple linear regression model accounted for 13.0% of the variance in future MDD symptoms, *F*(6, 1047) = 27.26, *p* < .001 (Table 3). Similarly, W2 MDD symptoms were most strongly predicted by MDD symptoms at W1 (*d* = 0.63, *p* < .001). However, the main effects of social support (*d* = −0.21, *p* < .001) and social strain (*d* = 0.22, *p* < .001) were found to maintain independent associations with future MDD symptoms, while the main effect of IL-6 remained nonsignificant (*d* = 0.02, *p* = .767; tests of H_1_). Additionally, the interaction term for IL-6 × social support was nonsignificant (*d* = −0.06, *p* = .312; tests of H_2_), while the interaction term for IL-6 × social strain remained significant (*d* = 0.12, *p* = .048; tests of H_3_).

**Table 3:** Multiple regression model of W1 MSD IL-6 levels, social support, and strain on W2 MDD symptoms.

**Linear model estimates**					
Parametric coefficients	*b*	*(SE)*	*t*	*p*	*d*

(Intercept)	0.879∗∗∗	(0.261)	3.373	< .001	0.208
W1 MDD Symptoms	0.277∗∗∗	(0.027)	10.168	< .001	0.628
W1 Social support	−0.027∗∗∗	(0.008)	−3.454	< .001	−0.213
W1 Social strain	0.022∗∗∗	(0.006)	3.603	< .001	0.223
W1 IL-6	0.124	(0.417)	0.296	.767	0.018
W1 Social support × IL-6	−0.012	(0.012)	−1.011	.312	−0.062
W1 Social strain × IL-6	0.018∗	(0.009)	1.977	.048	0.122

$R^{2}$	0.135				
Adjusted $R^{2}$	0.130				
*F*-statistic	27.26∗∗∗				
*p*	< .001				

**Generalized additive model (GAM) non-linear estimates**					
Parametric coefficients	*b*	*(SE)*	*t*	*p*	*d*

(Intercept)	–	(−)	–	–	–
W1 MDD Symptoms	0.273∗∗∗	(0.027)	10.171	< .001	0.629
W1 Social support	−0.052∗∗	(0.017)	−3.062	.002	−0.189
W1 Social strain	0.091∗∗∗	(0.026)	3.485	< .001	0.215
W1 IL-6	–	(−)	–	–	–

Significance of smooth terms	*edf*	*rdf*	*F*	*p*	

s(W1 Social support × IL-6)	9.536	12.395	1.49	.092	
s(W1 Social strain × IL-6)	6.240	8.778	2.94∗∗	.002	

Rank	59/62				
Adjusted $R^{2}$	0.171				
Deviance explained	18.50%				

*Note*. ∗*p* < .05, ∗∗*p* < .01, ∗∗∗*p* < .001.

W1 = wave 1; MSD = Meso Scale Discovery; IL-6 = interleukin-6; W2 = wave 2; MDD = major depressive disorder; *edf* = estimated degrees of freedom; *rdf* = reference degrees of freedom; *F* = *F*-ratio statistic.

s() = smoothed terms to accommodate any non-linear relations.

**Fig. 3:**
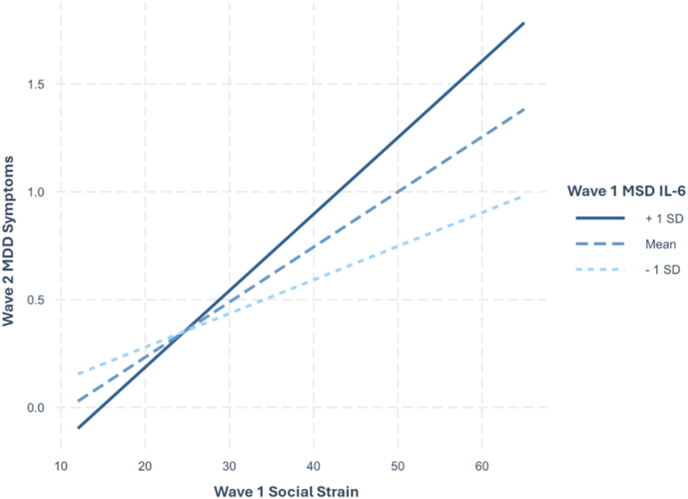
Simple Slopes of W1 Social Strain predicting W2 MDD Symptoms at Levels of MSD IL-6 *Note*. W1 = wave 1; W2 = wave 2; MDD = Major depressive disorder; MSD = Meso Scale Discovery immunoassay, IL-6 = interleukin-6.

Follow-up simple slope analyses based on low (*x* ≤ −0.387), medium (−0.387 < *x* < 0.734), and high (0.734 ≤ *x*) log-transformed values of IL-6 were conducted. Corroborating previous results, social strain predicted future MDD symptoms at high (*d* = 0.29, *p* < .001) and medium (*d* = 0.27, *p* < .001) levels of IL-6, but not at low levels (*d* = 0.21, *p* = .050) (Fig. 3). Generalized additive models fully corroborate these findings when examining non-linear associations, with IL-6 × social strain remaining significant and IL-6 × social support remaining nonsignificant (Table 3 and Fig. S2).

**Covariate-adjusted analyses.** All analyses were then re-run while adjusting for potential covariates, including treatment exposure in the past 12 months (visits to medical doctors and visits to mental health professionals, respectively), body mass index (BMI), smoking history, the number of chronic conditions, and medication use at W1. The OSM details these covariate-adjusted analyses. Although these covariates contributed meaningfully to the prediction of W2 MDD symptoms, they did not change the pattern of associations found in our original predictor or interaction terms when adjusting for the full set of covariates (Table S2–3). However, when the model was adjusted solely for treatment exposure covariates (Tables S4-5), the interaction effect between ELISA IL-6 and social support was attenuated in the GAM model, where it did not reach statistical significance.

### 3.3 Moderated mediation analyses

Additionally, moderated mediation analyses were conducted to examine whether the moderating influence of IL-6 on future MDD symptoms was mediated by C-reactive protein (CRP) or fibrinogen levels (Table S6–7). These additional analyses were based on emerging evidence implicating CRP and fibrinogen as downstream markers of inflammatory processes shaped by the social context (Zainal, 2025). Although these mediation models were estimated across two waves rather than the recommended three for establishing longitudinal mediation (Maxwell and Cole, 2007), they provide preliminary evidence for plausible pathways linking social context, inflammation, and MDD symptoms.

**Moderated mediation analyses for ELISA IL-6.** CRP and fibrinogen were tested as potential mediators of the interactive effects found with ELISA IL-6 levels (Table S6). ELISA IL-6 was positively associated with both CRP (*b* = 0.202, *p* < .001) and fibrinogen levels (*b* = 0.199, *p* < .001). While controlling for ELISA IL-6 levels, fibrinogen (*b* = 1.520, *p* = .210) and its interaction terms fibrinogen × social support (*b* = −0.060, *p* = .002), and fibrinogen × social strain (*b* = 0.054, *p* < .001) significantly predicted future MDD symptoms, while CRP (*b* = −1.314, *p* = .072), CRP × social support (*b* = 0.031, *p* = .147), and CRP × social strain (*b* = −0.003, *p* = .845) did not significantly predict future MDD symptoms.

After accounting for these mediators, the direct effect of ELISA IL-6 × social support (*b* = −0.020, *p* = .086) was no longer significant, while the effect of ELISA IL-6 × social strain (*b* = 0.020, *p* = .037) remained significant. Joint mediation by CRP and fibrinogen levels accounted for 21.0% of the total effect of the ELISA IL-6 × social support interaction (indirect effect = −0.005, 95% CI [−0.021, 0.007] and 32.7% of the ELISA IL-6 × social strain interaction (indirect effect = 0.010, 95% CI [−0.001, 0.021], though these mediation effects remained nonsignificant. Component-wise mediation analyses showed that fibrinogen (*b* = −0.010) had a stronger mediation effect than CRP (*b* = −0.001) for ELISA IL-6 × social support. Similarly, fibrinogen levels (*b* = 0.010) had a stronger mediation effect than CRP (*b* = 0.005) for ELISA IL-6 × social strain. Lastly, the full mediation model including CRP, fibrinogen, and their interactions with social support and strain significantly improved model fit relative to the baseline model, *F*(6, 1039) = 4.46, *p* < .001.

**Moderated mediation analyses for MSD IL-6.** Parallel analyses using MSD IL-6 levels revealed similar findings (Table S7). MSD IL-6 levels were positively associated with both CRP (*b* = 0.197, *p* < .001) and fibrinogen (*b* = 0.188, *p* < .001) levels. While controlling for 10.13039/100030732MSD IL-6, fibrinogen levels (*b* = 1.619, *p* = .014) and its interaction terms fibrinogen × social support (*b* = −0.063, *p* = .001), and fibrinogen × social strain (*b* = 0.056, *p* < .001) significantly predicted future MDD symptoms, while CRP levels (*b* = −1.020, *p* = .158), CRP × social support (*b* = 0.022, *p* = .305), and CRP × social strain (*b* = 0.003, *p* = .853) were not significant predictors.

After accounting for these mediator variables, the direct effect of 10.13039/100030732MSD IL-6 × social strain on W2 MDD severity (*b* = 0.010, *p* = .281) was no longer significant, and the effect of 10.13039/100030732MSD IL-6 × social support remained nonsignificant (*b* = −0.005, *p* = .669). The joint mediation by CRP and fibrinogen levels accounted for 63.4% of the total effect of 10.13039/100030732MSD IL-6 × social support effect and 48.9% of the IL-6 × social strain effect, with the mediation of 10.13039/100030732MSD IL-6 × social strain being significant (indirect effect = 0.010, 95% CI [−0.001, 0.027]). In contrast, the mediation of 10.13039/100030732MSD IL-6 × social support by CRP and fibrinogen (indirect effect = −0.009, 95% CI [−0.037, 0.003] was nonsignificant. Component-wise mediation analyses showed that fibrinogen levels (*b* = −0.012) had a stronger mediation effect than CRP levels (*b* = −0.002) for 10.13039/100030732MSD IL-6 × social support. Similarly, fibrinogen (*b* = 0.010) had a stronger mediation effect than CRP levels (*b* = 0.004) for MSD IL-6 × social strain. Lastly, the full mediation model including CRP, fibrinogen, and their interactions with social support and strain significantly improved model fit over the baseline model, *F*(6, 1039) = 5.30, *p* < .001.

### 3.4 Exploring the depression-to-inflammation pathway

Sensitivity analysis examined whether W1 predictors accounted for variability in IL-6 levels at W2 (Tables S8-9), adjusting for all covariates. Findings indicated no significant link between W1 MDD severity and W2 ELISA IL-6 levels (*d* = 0.002, *p* = .833) or W2 MSD IL-6 levels (*d* = 0.004, *p* = .658).

## 4 Discussion

The present study examined the interactions between IL-6 levels, social support, and social strain in predicting future MDD symptoms. The findings suggested that higher IL-6 levels strengthened the relationship between lower social support and higher social strain in predicting greater nine-year MDD severity. Importantly, these patterns remained robust after controlling for baseline MDD severity and various covariates, highlighting their plausible prognostic value. Collectively, these outcomes emphasize the possibility of a unified course where proinflammatory activity and interpersonal risk factors jointly shape MDD symptom trajectories.

In the preliminary analyses, the absence of significant change in MDD symptoms from W1 to W2 indicated minimal group-level variability. Although both social support and social strain increased significantly across waves, they were small in magnitude and not reliably associated across assays, suggesting negligible changes at the population level. At W1, ELISA IL-6 levels were positively associated with both social support and strain, suggesting that higher inflammation may be related to greater social involvement overall rather than specifically to negative or positive social exchanges. The lack of notable associations for MSD IL-6 levels and social support or strain aligns with previous reports of assay-based differences in IL-6 (Leng et al., 2008). Although there is intuitive appeal in the idea that higher inflammation would correspond with greater social strain and less support, the observed associations at W1 herein may reflect the complexity of social dynamics. For instance, individuals with larger social networks often experience both more supportive and more strained interactions (Shin and Gyeong, 2023). These findings may also be shaped by the influence of other moderator-level influences, such as perceived support giving (Jiang et al., 2022), which has previously explained differential associations between inflammation and social support in the MIDUS cohort. Collectively, these initial analyses offer context in the interpretation of the prospective links among inflammation, social support, and MDD symptoms.

Regarding our hypotheses, when accounting for IL-6 and hypothesized interactions, social support and strain did not consistently show independent associations with nine-year MDD symptoms across IL-6 assay methods, contrary to H_1_. Social support notably interacted with IL-6 levels when measured through ELISA, but not MSD (partial support for H_2_). In contrast, social strain significantly interacted with IL-6 levels across both methods of quantification (full support for H_3_). In both interactions, higher levels of IL-6 served as a biological vulnerability, amplifying the effects of lower social support and higher social strain in predicting future MDD symptoms. Simple slope analyses further revealed that lower social support was significantly associated with future MDD symptoms at intermediate and high levels of IL-6, but not at low levels. This pattern is consistent with and extends the buffering hypothesis (Cohen and Wills, 1985), which posits that social support attenuates the physiological consequences of stress, including those linked to inflammation. At higher IL-6 levels, the buffering effect of social support may be especially pronounced, alleviating inflammation-induced disruptions in neurobiological processes implicated in depression, such as HPA axis dysregulation (Hassamal, 2023) and serotonin depletion (Zhang et al., 2023). By contrast, at lower IL-6 levels, the pathological processes underlying inflammation-induced MDD symptoms may be less pronounced, thereby diminishing the buffering effect of social support. These outcomes extend prior research, suggesting that social support not only confers psychosocial benefits but may also attenuate the physiological impact of proinflammatory challenges (Friuli et al., 2021), such as by engaging the oxytocinergic pathways that reduce cortisol secretion.

Similarly, social strain predicted future MDD symptoms at intermediate and high levels of IL-6, but not at low levels. This pattern suggests that the stress resulting from strained social relationships may be more detrimental under conditions of heightened inflammation (Yang et al., 2014). Social strain reflects chronic exposure to interpersonal and related social stressors, which can exacerbate proinflammatory processes and contribute to the onset and persistence of MDD symptoms over time (Yang et al., 2014). Our results suggest that when inflammation levels are high, individuals may be more vulnerable to the detrimental effects of social strain (Kopschina Feltes et al., 2017). Mechanistically, chronic social strain may intensify the proinflammatory response by increasing blood-brain barrier (BBB) permeability (Wang and Russo, 2024) and potentiating the noradrenergic response (Seki et al., 2018). These factors could perpetuate a cycle of stress and inflammation that increases the risk for depression (Shin and Gyeong, 2023).

Simultaneously, IL-6 moderated several pathways linking social support and strain to MDD symptoms nine years later, mostly with small effect sizes. Although the observed interaction effect sizes were modest (*d* = 0.13 to 0.18) and below conventional thresholds for small effects, they are consistent with prior research examining psychosocial influences on inflammation in heterogeneous, non-clinical populations (Miller et al., 2011). Longitudinal studies similarly reported small effect sizes in the range of *d* = 0.1 to 0.2 (Kiecolt-Glaser et al., 2015), reflecting the complex interplay of social dynamics and biological processes influencing inflammation. Importantly, even subtle variations in inflammatory markers such as IL-6 have been linked to meaningful changes in health trajectories and a heightened risk of chronic disease (Marsland et al., 2017b). Considering our study's extended follow-up period and the deployment of two distinct IL-6 assays, these modest interaction effects are likely to represent biologically and clinically relevant processes, underscoring the practical significance of psychosocial modulation of inflammation.

These results and interpretations should be considered in light of several limitations. First, the reliance on a community-dwelling adult sample may limit the generalizability of the findings to other age groups or clinical populations. The relevance of the current findings to specific clinical or developmental challenges, such as treatment resistance (Yang et al., 2019) and the presence of comorbid psychiatric disorders (Platona et al., 2024), warrants further investigation. Future studies should include more diverse populations to determine whether these patterns persist in broader demographic or clinical contexts.

Second, while the study's focus on the role of IL-6 as a broad marker of peripheral proinflammatory activity (Roohi et al., 2021) provides a valuable entry point, IL-6 alone may not capture the full complexity of the inflammatory profile involved in depression or its inflammatory subtype, which involves additional cytokines such as IL-1β and TNF-α (Ahmed, 2011). Consistent with recent findings, IL-6 levels were robustly correlated with fibrinogen and CRP levels in this study. Furthermore, we observed that fibrinogen consistently mediated the moderating effects of IL-6 on the social strain pathway leading to future MDD symptoms. This suggests that evaluating IL-6 vis-à-vis fibrinogen and CRP may better capture nuanced pathways through which inflammation interacts with psychosocial factors to influence depression symptom risk. Thus, IL-6 alone may not fully capture systemic immune activation or the distinct immune pathways related to MDD symptom clusters (Zeng et al., 2023). Further studies should explore broader inflammatory panels, beyond fibrinogen and CRP, to better characterize these immunological signatures within psychosocial contexts and refine the mechanistic understanding of inflammation in depression (Gabay, 2006; Kaplanski et al., 2003).

Third, other limitations pertain to the study design and analytic approach. The study's two-wave design may preclude definite conclusions about the interactions between social support, strain, and inflammation on MDD symptoms over time (Rohrer and Murayama, 2023; Tennant et al., 2022). Future longitudinal studies with repeated, consistent measures would provide a clearer understanding of these relationships and refine conclusions about their temporal sequence (Schober and Vetter, 2018). It also remains plausible that other factors, such as genetic predispositions, hormonal influences, and neurobiological markers (Menke, 2024; Miller et al., 2009), may also modulate the depressogenic effects of inflammation. Future research should investigate the combined effects and interactions of such factors and identify specific pathways that shape the risk of depression and its inflammatory subtype (Himmerich et al., 2019). Recent studies also suggest differential associations between various markers of inflammation and specific MDD symptoms (Zeng et al., 2023). Future studies should address potential heterogeneities in symptom presentation, such as age (Schaakxs et al., 2017) and sex-related (Thompson et al., 2021) variabilities. Lastly, machine learning techniques are well-suited for handling multivariable analyses on high-dimensional datasets, allowing for the examination of various proinflammatory markers and potential confounders that may precede the incidence or recurrence of MDD symptoms (Yarkoni and Westfall, 2017; Zainal and Van Doren, 2025).

Despite these limitations, the study is supported by several strengths. These results are based on longitudinal data collected over a nine-year period from an established and well-characterized nationally representative sample (Ryff et al., 2019). Moreover, the sensitivity analyses considered a broad range of sociodemographic and health-related factors, thereby enhancing the validity of the observed associations. Lastly, we examined both the independent and interactive effects of social support, strain, and inflammation, offering insights into their relative contributions in predicting MDD symptoms over an extended period.

The findings from the present study carry important clinical implications if replicated in future studies. Interventions aimed at increasing social support or reducing social strain may be especially relevant in the context of elevated inflammation, such as those with underlying systemic proinflammatory conditions (Irwin et al., 2019; Kuhlman et al., 2020). 10.13039/100014337Furthermore, strategies addressing either social support or social strain should consider both aspects in alleviating the psychosocial risk for inflammation-induced depression in light of these findings (Shin and Gyeong, 2023). Our study also examined the independent and interactive effects of IL-6 concentrations, social support, and social strain on future MDD symptoms, utilizing two IL-6 quantification platforms: ELISA and MSD. Differences in findings between these platforms are not unexpected, given their distinct analytical characteristics (Lasseter et al., 2020). ELISA assays, which are extensively used in cytokine research, offer high specificity but typically operate within a narrower dynamic range and may be less sensitive to extremely low or high IL-6 concentrations. In contrast, MSD assays provide a broader dynamic range, which enhances sensitivity and expands the dynamic range, reducing floor and ceiling effects. However, it may be less sensitive to physiological processes localized within specific concentration intervals (Thompson et al., 2012). Moreover, biomarker-psychosocial interactions are inherently subtle and are subject to the sensitivity profiles of each test. Similar discrepancies have been documented in prior work, reinforcing the value of cross-validating biomarker findings across multiple assay platforms to enhance robustness and replicability (Leng et al., 2008). Overall, this work underscores the importance of deploying multiple assay platforms to provide complementary insights and a reliable understanding of biomarker-behavior relationships.

To summarize, our study examined the role of serum IL-6, social support, and social strain in predicting future MDD symptoms over a nine-year period. Specifically, we tested the hypothesis that elevated inflammation exacerbates the influence of psychosocial risk factors on the onset and development of MDD symptoms. Consistent with research suggesting interactions between inflammation and psychosocial stressors, our findings provide preliminary support for dynamic vulnerability models, such as various two-hit models of depression (Irwin and Piber, 2018). In individuals with first-hit exposures, such as prolonged inflammation from systemic illness, an examination of existing second-hit stressors, such as poor social support and high social strain, is crucial for understanding their susceptibility to developing depression. Levels of inflammation were found to act as a biological vulnerability, amplifying the effects of both lower social support and higher strain on future MDD symptoms. Further research should investigate the mechanistic pathways linking inflammation and specific psychosocial factors (Eisenberger et al., 2017) and explore how these interactions may inform targeted interventions addressing both biological and social determinants of depression. Although current evidence suggests that systems such as oxytocin signaling (Walker et al., 2020), HPA axis regulation (Hassamal, 2023), and blood-brain barrier permeability (Welcome, 2020) may play roles in shaping individual responses to social and emotional stressors, the relevance of these mechanisms and whether they mediate the findings of this study warrant further empirical investigation. Exploring these pathways may ultimately help clarify how biological and psychosocial processes converge to influence MDD symptoms and could inform the development of targeted interventions that address both the physiological and sociocultural determinants of mental health.

## CRediT authorship contribution statement

**Timothy Bang Hao Aw:** Writing – review & editing, Writing – original draft, Validation, Investigation, Data curation, Conceptualization. **Nur Hani Zainal:** Writing – review & editing, Writing – original draft, Visualization, Validation, Supervision, Methodology, Investigation, Funding acquisition, Formal analysis, Data curation, Conceptualization.

## Funding source(s)

Assistant Professor Zainal received funding support from the 10.13039/501100001352National University of Singapore (NUS) Presidential Young Professorship (PYP) Start-Up Grant (SUG) and White Space Fund from July 1, 2024, to June 30, 2029. Since 1995, the MIDUS study has been funded by the following sources: the John D. and Catherine T. MacArthur Foundation Research Network and the National Institute on Aging (P01-AG020166; U19-AG051426).

## Declaration of competing interest

The authors declare that they have no known competing financial interests or personal relationships that could have appeared to influence the work reported in this paper.

## Data Availability

The authors do not have permission to share data.
