# Supplementary material for: Interleukin-6 moderates the relationship between social support, strain, and future depressive symptoms
Source: Brain Behav Immun Health. 2025 Oct 13;49:101122. doi: 10.1016/j.bbih.2025.101122 (PMC12590439; doi:10.1016/j.bbih.2025.101122)
Supplement: Multimedia component 1 [file mmc1.docx]

# Table S1

## Pearson Correlations Between IL-6, Social Support, and Social Strain at W1

| Variable | | *M* | (*SD*) | 1 | 2 | 3 | 4 |
| --- | --- | --- | --- | --- | --- | --- | --- |
| 1 | ELISA IL-6^a^ | 0.70 | (0.65) | - |  |  |  |
| 2 | MSD IL-6^a^ | 0.17 | (0.56) | .58*** | - |  |  |
| 3 | Social Support | 46.86 | (10.08) | .09** | .03 | - |  |
| 4 | Social Strain | 30.51 | (12.73) | .09** | .05 | .74*** | - |

*Note*. ***p* < .01, ****p* < .001.

IL-6 = interleukin-6; W1 = wave 1; ELISA = enzyme-linked immunosorbent assay; MSD = Meso Scale Discovery assay.

^a^Log-transformed values.

# Table S2

## Covariate-Adjusted Models of W1 ELISA IL-6 Levels, Social Support, and Strain with All Relevant Covariates on W2 MDD Symptoms

| **Linear model estimates** | | | | | |
| --- | --- | --- | --- | --- | --- |
| Parametric coefficients | *b* | *(SE)* | *t* | *p* | *d* |
| (Intercept) | -0.014 | (0.461) | -0.031 | .975 | -0.002 |
| W1 MDD Symptoms | 0.021*** | (0.028) | 7.482 | < .001 | 0.464 |
| W1 Social support | -0.008 | (0.011) | -0.735 | .462 | -0.046 |
| W1 Social strain | 0.001 | (0.009) | 0.163 | .871 | 0.010 |
| W1 IL-6 | 0.472 | (0.366) | 1.288 | .198 | 0.080 |
| Medical treatment visits | -0.003 | (0.013) | -0.269 | .788 | -0.017 |
| Mental health treatment visits | 0.028*** | (0.006) | 4.861 | < .001 | 0.301 |
| Body mass index | 0.014 | (0.009) | 1.550 | .121 | 0.096 |
| History of smoking | -0.075 | (0.098) | -0.763 | .446 | -0.047 |
| Number of chronic conditions | 0.012 | (0.025) | 0.472 | .637 | 0.029 |
| Medication use | 0.140*** | (0.039) | 3.636 | < .001 | 0.225 |
| W1 Social support × IL-6 | -0.025* | (0.011) | -2.379 | .018 | -0.147 |
| W1 Social strain × IL-6 | 0.027** | (0.009) | 3.198 | .001 | 0.198 |
| *R^2^* | 0.182 |  |  |  |  |
| Adjusted *R^2^* | 0.173 |  |  |  |  |
| *F*-statistic | 19.36*** |  |  |  |  |
| *p* | < .001 |  |  |  |  |
| **Generalized additive model (GAM) non-linear estimates** | | | | | |
| Parametric coefficients | *b* | *(SE)* | *t* | *p* | *d* |
| (Intercept) | 0.348 | (0.373) | 0.932 | .352 | 0.058 |
| W1 MDD Symptoms | 0.213*** | (0.028) | 7.515 | < .001 | 0.466 |
| W1 Social support | -0.025** | (0.007) | -3.440 | < .001 | -0.213 |
| W1 Social strain | 0.020** | (0.006) | 3.373 | < .001 | 0.209 |
| W1 IL-6 | 0.714*** | (0.182) | 3.923 | < .001 | 0.243 |
| Medical treatment visits | -0.005 | (0.013) | -0.357 | .721 | -0.022 |
| Mental health treatment visits | 0.030*** | (0.006) | 5.072 | < .001 | 0.314 |
| Body mass index | 0.013 | (0.009) | 1.396 | .163 | 0.087 |
| History of smoking | -0.078 | (0.097) | -0.805 | .421 | -0.050 |
| Number of chronic conditions | 0.009 | (0.025) | 0.371 | .710 | 0.023 |
| Medication use | 0.140*** | (0.038) | 3.632 | < .001 | 0.225 |
| Significance of smooth terms | *edf* | *rdf* | *F* | *p* |  |
| s(W1 Social support × IL-6) | 1.000 | 1.000 | 5.058* | .025 |  |
| s(W1 Social strain × IL-6) | 3.011 | 3.524 | 5.390*** | .001 |  |
| Rank | 19/21 |  |  |  |  |
| Adjusted *R^2^* | 0.180 |  |  |  |  |
| Deviance explained | 19.10% |  |  |  |  |

*Note*. **p* < .05, ***p* < .01, ****p* < .001.

W1 = wave 1; ELISA = enzyme-linked immunosorbent assay; IL-6 = interleukin-6; W2 = wave 2; MDD = major depressive disorder; *edf* = estimated degrees of freedom; *rdf* = reference degrees of freedom; *F* = *F*-ratio statistic.

s() = smoothed terms to accommodate any nonlinear relations.

# Table S3

## Covariate-Adjusted Models of W1 MSD IL-6 Levels, Social Support, and Strain with All Relevant Covariates on W2 MDD Symptoms

| **Linear model estimates** | | | | | |
| --- | --- | --- | --- | --- | --- |
| Parametric coefficients | *b* | *(SE)* | *t* | *p* | *d* |
| (Intercept) | 0.201 | (0.370) | 0.544 | .587 | 0.034 |
| W1 MDD Symptoms | 0.214*** | (0.028) | 7.536 | < .001 | 0.467 |
| W1 Social support | -0.022** | (0.008) | -2.938 | .003 | -0.182 |
| W1 Social strain | 0.017** | (0.006) | 2.761 | .006 | 0.171 |
| W1 IL-6 | 0.214 | (0.410) | 0.523 | .601 | 0.032 |
| Medical treatment visits | -0.004 | (0.013) | -0.292 | .771 | -0.018 |
| Mental health treatment visits | 0.027*** | (0.006) | 4.647 | < .001 | 0.288 |
| Body mass index | 0.015 | (0.009) | 1.704 | .089 | 0.106 |
| History of smoking | -0.076 | (0.098) | -0.777 | .438 | -0.048 |
| Number of chronic conditions | 0.019 | (0.025) | 0.741 | .459 | 0.046 |
| Medication use | 0.147*** | (0.039) | 3.784 | < .001 | 0.235 |
| W1 Social support × IL-6 | -0.018 | (0.012) | -1.533 | .126 | -0.095 |
| W1 Social strain × IL-6 | 0.022* | (0.009) | 2.487 | .013 | 0.154 |
| *R^2^* | 0.178 |  |  |  |  |
| Adjusted *R^2^* | 0.169 |  |  |  |  |
| *F*-statistic | 18.82*** |  |  |  |  |
| *p* | < .001 |  |  |  |  |
| **Generalized additive model (GAM) non-linear estimates** | | | | | |
| Parametric coefficients | *b* | *(SE)* | *t* | *p* | *d* |
| (Intercept) | 0.240 | (0.368) | 0.653 | .514 | 0.040 |
| W1 MDD Symptoms | 0.213*** | (0.028) | 7.525 | < .001 | 0.466 |
| W1 Social support | -0.022** | (0.008) | -2.923 | .004 | -0.181 |
| W1 Social strain | 0.017** | (0.006) | 2.752 | .006 | 0.171 |
| Medical treatment visits | -0.003 | (0.013) | -0.254 | .800 | -0.016 |
| Mental health treatment visits | 0.028*** | (0.006) | 4.815 | < .001 | 0.298 |
| Body mass index | 0.014 | (0.009) | 1.607 | .108 | 0.100 |
| History of smoking | -0.084 | (0.097) | -0.861 | .389 | -0.053 |
| Number of chronic conditions | 0.015 | (0.025) | 0.595 | .552 | 0.037 |
| Medication use | 0.141*** | (0.039) | 3.656 | < .001 | 0.227 |
| W1 IL-6 | -10.410** | (3.379) | -3.081 | .002 | -0.191 |
| Significance of smooth terms | *edf* | *rdf* | *F* | *p* |  |
| s(W1 Social support × IL-6) | 2.563 | 3.193 | 1.976 | .158 |  |
| s(W1 Social strain × IL-6) | 5.398 | 6.54 | 2.986** | .004 |  |
| Rank | 29/31 |  |  |  |  |
| Adjusted *R^2^* | 0.185 |  |  |  |  |
| Deviance explained | 19.90% |  |  |  |  |

*Note*. **p* < .05, ***p* < .01, ****p* < .001.

W1 = wave 1; MSD = Meso Scale Discovery assay; IL-6 = interleukin-6; W2 = wave 2; MDD = major depressive disorder; *edf* = estimated degrees of freedom; *rdf* = reference degrees of freedom; *F* = *F*-ratio statistic.

s() = smoothed terms to accommodate any nonlinear relations.

# Table S4A

## Multiple Regression Model of W1 ELISA IL-6 Levels, Social Support, Strain, and Treatment Visits on W2 MDD Symptoms

| **Linear model estimates** | | | | | |
| --- | --- | --- | --- | --- | --- |
| Parametric coefficients | *b* | *(SE)* | *t* | *p* | *d* |
| (Intercept) | 0.526 | (0.373) | 1.409 | .159 | 0.087 |
| W1 MDD Symptoms | 0.233*** | (0.028) | 8.335 | < .001 | 0.517 |
| W1 Social support | -0.010 | (0.011) | -0.892 | .373 | -0.055 |
| W1 Social strain | 0.002 | (0.009) | 0.185 | .853 | 0.011 |
| W1 IL-6 | 0.494 | (0.369) | 1.338 | .181 | 0.083 |
| Medical treatment visits | 0.012 | (0.012) | 0.952 | .341 | 0.059 |
| Mental health treatment visits | 0.029*** | (0.006) | 4.981 | < .001 | 0.309 |
| W1 Social support × IL-6 | -0.025* | (0.011) | -2.364 | .018 | -0.147 |
| W1 Social strain × IL-6 | 0.029*** | (0.009) | 3.364 | < .001 | 0.209 |
| *R^2^* | 0.164 |  |  |  |  |
| Adjusted *R^2^* | 0.158 |  |  |  |  |
| *F*-statistic | 25.62*** |  |  |  |  |
| *p* | < .001 |  |  |  |  |
| **Generalized additive model (GAM) non-linear estimates** | | | | | |
| Parametric coefficients | *b* | *(SE)* | *t* | *p* | *d* |
| (Intercept) | - | (-) | - | - | - |
| W1 MDD Symptoms | 0.238*** | (0.028) | 8.593 | < .001 | 0.535 |
| W1 Social support | -0.044** | (0.016) | -2.770 | .006 | -0.173 |
| W1 Social strain | 0.076** | (0.024) | 3.140 | .002 | 0.196 |
| Medical treatment visits | 0.008 | (0.012) | 0.623 | .533 | 0.039 |
| Mental health treatment visits | 0.025*** | (0.006) | 4.299 | < .001 | 0.268 |
| W1 IL-6 | - | (-) | - | - | - |
| Significance of smooth terms | *edf* | *rdf* | *F* | *p* |  |
| s(W1 Social support × IL-6) | 7.980 | 10.521 | 1.674 | .076 |  |
| s(W1 Social strain × IL-6) | 5.848 | 8.385 | 2.524** | .010 |  |
| Rank | 61/64 |  |  |  |  |
| Adjusted | 0.183 |  |  |  |  |
| Deviance explained | 19.7% |  |  |  |  |

*Note*. **p* < .05, ***p* < .01, ****p* < .001.

W1 = wave 1; ELISA = enzyme-linked immunosorbent assay; IL-6 = interleukin-6; W2 = wave 2; MDD = major depressive disorder; *edf* = estimated degrees of freedom; *rdf* = reference degrees of freedom; *F* = *F*-ratio statistic.

s() = smoothed terms to accommodate any nonlinear relations.

# Table S4B

## Model Comparison of Original and Treatment-Adjusted Models of ELISA IL-6 on W1 Social Support and Strain Pathways Predicting W2 MDD Symptoms

| Model | *Adj. R^2^* | *df* | *F* | *p* |
| --- | --- | --- | --- | --- |
| 1: Baseline | 0.136 |  |  |  |
| 2: Full Mediation | 0.158 | 2 | 14.33*** | < .001 |

*Note*. ****p* < .001

ELISA = enzyme-linked immunosorbent assay; IL-6 = interleukin-6; W1 = wave 1; W2 = wave 2; MDD = major depressive disorder; *Adj. R^2^* = adjusted *R*-squared; *F* = *F*-ratio statistic.

Model 1 includes the predictors: W1 MDD symptoms, social support, social strain, and IL-6 interaction terms. Model 2 adds the treatment visit terms.

# Table S5A

## Multiple Regression Model of W1 MSD IL-6 Levels, Social Support, Strain, and Treatment Visits on W2 MDD Symptoms

| **Linear model estimates** | | | | | |
| --- | --- | --- | --- | --- | --- |
| Parametric coefficients | *b* | *(SE)* | *t* | *p* | *d* |
| (Intercept) | 0.794** | (0.262) | 3.027 | .003 | 0.187 |
| W1 MDD Symptoms | 0.239*** | (0.028) | 8.555 | < .001 | 0.529 |
| W1 Social support | -0.025** | (0.008) | -3.223 | .001 | -0.199 |
| W1 Social strain | 0.019** | (0.006) | 3.107 | .002 | 0.192 |
| W1 IL-6 | 0.188 | (0.413) | 0.455 | .649 | 0.028 |
| Medical treatment visits | 0.014 | (0.012) | 1.091 | .276 | 0.067 |
| Mental health treatment visits | 0.028*** | (0.006) | 4.700 | < .001 | 0.291 |
| W1 Social support × IL-6 | -0.015 | (0.012) | -1.240 | .215 | -0.077 |
| W1 Social strain × IL-6 | 0.020* | (0.009) | 2.229 | .026 | 0.138 |
| *R^2^* | 0.156 |  |  |  |  |
| Adjusted *R^2^* | 0.150 |  |  |  |  |
| *F*-statistic | 24.19*** |  |  |  |  |
| *p* | < .001 |  |  |  |  |
| **Generalized additive model (GAM) non-linear estimates** | | | | | |
| Parametric coefficients | *b* | *(SE)* | *t* | *p* | *d* |
| (Intercept) | - | (-) | - | - | - |
| W1 MDD Symptoms | 0.241*** | (0.028) | 8.707 | < .001 | 0.542 |
| W1 Social support | -0.046** | (0.016) | -2.833 | .005 | -0.176 |
| W1 Social strain | 0.079** | (0.025) | 3.190 | .001 | 0.199 |
| Medical treatment visits | 0.009 | (0.012) | 0.737 | .461 | 0.046 |
| Mental health treatment visits | 0.024*** | (0.006) | 4.147 | < .001 | 0.258 |
| W1 IL-6 | - | (-) | - | - | - |
| Significance of smooth terms | *edf* | *rdf* | *F* | *p* |  |
| s(W1 Social support × IL-6) | 8.685 | 11.324 | 1.350 | .199 |  |
| s(W1 Social strain × IL-6) | 5.799 | 8.227 | 2.625** | .007 |  |
| Rank | 61/64 |  |  |  |  |
| Adjusted | 0.182 |  |  |  |  |
| Deviance explained | 19.6% |  |  |  |  |

*Note*. ***p* < .01, ****p* < .001.

W1 = wave 1; MSD = Meso Scale Discovery assay; IL-6 = interleukin-6; W2 = wave 2; MDD = major depressive disorder; *edf* = estimated degrees of freedom; *rdf* = reference degrees of freedom; *F* = *F*-ratio statistic.

s() = smoothed terms to accommodate any nonlinear relations.

# Table S5B

## Model Comparison of Original and Treatment-Adjusted Models of W1 MSD IL-6 on Social Support and Strain Pathways Predicting W2 MDD Symptoms

| Model | *Adj. R^2^* | *df* | *SS* | *F* | *p* |
| --- | --- | --- | --- | --- | --- |
| 1: Baseline | 0.130 |  |  |  |  |
| 2: Full Mediation | 0.150 | 2 | 65.74 | 13.10*** | < .001 |

*Note*. ****p* < .001.

W1 = wave 1; MSD = Meso Scale Discovery assay; IL-6 = interleukin-6; W2 = wave 2; MDD = major depressive disorder; *Adj. R^2^* = adjusted *R*-squared; *F* = *F*-ratio statistic.

Model 1 includes the predictors: W1 MDD symptoms, social support, social strain, and IL-6 interaction terms. Model 2 adds the treatment visit terms.

# Table S6A

## Moderated Mediation Analysis of W1 ELISA IL-6 on Social Support and Strain Pathways on W2 W2 MDD Symptoms in Treatment-Adjusted Models

| *Pathway* | | *b* | (*SE*) | *t* | *p* |
| --- | --- | --- | --- | --- | --- |
| IL-6 Total Effects | |  |  |  |  |
|  | IL-6 × Social Support | -0.025* | (0.011) | -2.364 | .018 |
|  | IL-6 × Social Strain | 0.029*** | (0.009) | 3.364 | < .001 |
| (Path A) IL-6 → Mediators | |  |  |  |  |
|  | IL-6 → CRP | 0.202*** | (0.017) | 11.982 | < .001 |
|  | IL-6 → Fibrinogen | 0.199*** | (0.019) | 10.216 | < .001 |
| (Path B) Mediators → Outcome | |  |  |  |  |
|  | CRP | -1.314 | (0.728) | -1.804 | .072 |
|  | CRP × Social Support | 0.031 | (0.021) | 1.453 | .147 |
|  | CRP × Social Strain | -0.003 | (0.017) | -0.195 | .845 |
|  | Fibrinogen | 1.520* | (0.657) | 2.312 | .021 |
|  | Fibrinogen × Social Support | -0.060** | (0.020) | -3.038 | .002 |
|  | Fibrinogen × Social Strain | 0.054*** | (0.015) | 3.551 | < .001 |
| (Path C) IL-6 Direct Effects | |  |  |  |  |
|  | IL-6 × Social Support | -0.020 | (0.012) | -1.720 | .086 |
|  | IL-6 × Social Strain | 0.020* | (0.009) | 2.089 | .037 |

*Note.* **p* < .05, ***p* < .01, ****p* < .001.

W1 = wave 1; ELISA = enzyme-linked immunosorbent assay; IL-6 = interleukin-6; W2 = wave 2; MDD = major depressive disorder; CRP = C-reactive protein.

# Table S6B

## Total and Mediation Effects of W1 ELISA IL-6 Moderation on Social Support and Strain Pathways Predicting W2 MDD Symptoms in Treatment-Adjusted Models

|  | |  | |  | |  | 95% Confidence Interval | |
| --- | --- | --- | --- | --- | --- | --- | --- | --- |
| Pathway | | *b* | % Total | | *p* | | Lower | Upper |
| IL-6 × Social Support | |  |  | |  | |  |  |
|  | Total Effects | -0.026* | 100% | | .018 | |  |  |
|  | Direct Effects | -0.020 | 79.0% | | .086 | |  |  |
|  | Mediation  (CRP + Fibrinogen)^a^ | -0.005 | 21.0% | | Not significant | | -0.021 | 0.007 |
|  | CRP only^b^ | -0.001 |  | |  | |  |  |
|  | Fibrinogen only^b^ | -0.010 |  | |  | |  |  |
| IL-6 × Social Strain | |  |  | |  | |  |  |
|  | Total Effects | 0.029*** | 100% | | < .001 | |  |  |
|  | Direct Effects | 0.020* | 66.3% | | .037 | |  |  |
|  | Mediation  (CRP + Fibrinogen)^a^ | 0.010 | 32.7% | | Not significant | | -0.001 | 0.021 |
|  | CRP only^b^ | 0.005 |  | |  | |  |  |
|  | Fibrinogen only^b^ | 0.010 |  | |  | |  |  |

*Note.* **p* < .05, ****p* < .001.

W1 = wave 1; ELISA = enzyme-linked immunosorbent assay; IL-6 = interleukin-6; W2 = wave 2; MDD = major depressive disorder; CRP = C-reactive protein.

^a^Bootstrapped confidence intervals.

^b^Component-wise mediation analyses.

# Table S6C

## Model Comparison of Baseline Moderation and Full Mediation of W1 ELISA IL-6 on Social Support and Strain Pathways Predicting W2 MDD Symptoms in Treatment-Adjusted Models

| Model | *Adj. R^2^* | *AIC* | *df* | *SS* | *F* | *p* |
| --- | --- | --- | --- | --- | --- | --- |
| 1: Baseline | 0.158 | 3961.9 |  |  |  |  |
| 2: Full Mediation | 0.174 | 3947.2 | 6 | 65.22 | 4.46*** | < .001 |

*Note*. ****p* < .001.

W1 = wave 1; ELISA = enzyme-linked immunosorbent assay; IL-6 = interleukin-6; W2 = wave 2; MDD = major depressive disorder; *Adj. R^2^* = adjusted *R*-squared; AIC = Akaike information criterion; *F* = *F*-ratio statistic.

Model 1 includes the predictors: W1 MDD symptoms, social support, social strain, IL-6 interaction terms, and treatment visit terms. Model 2 adds CRP, fibrinogen, and their interactions with social support and social strain.

# Table S7A

## Moderated Mediation Analysis of W1 MSD IL-6 on Social Support and Strain Pathways on W2 MDD Symptoms in Treatment-Adjusted Models

| *Pathway* | | *b* | (*SE*) | *t* | *p* |
| --- | --- | --- | --- | --- | --- |
| IL-6 Total Effects | |  |  |  |  |
|  | IL-6 × Social Support | -0.015 | (0.012) | -1.240 | .215 |
|  | IL-6 × Social Strain | 0.020* | (0.009) | 2.229 | .026 |
| (Path A) IL-6 → Mediators | |  |  |  |  |
|  | IL-6 → CRP | 0.197*** | (0.020) | 9.956 | < .001 |
|  | IL-6 → Fibrinogen | 0.188*** | (0.028) | 8.238 | < .001 |
| (Path B) Mediation Effects | |  |  |  |  |
|  | CRP | -1.020 | (0.721) | -1.414 | .158 |
|  | CRP × Social Support | 0.022 | (0.021) | 1.026 | .305 |
|  | CRP × Social Strain | 0.003 | (0.017) | 0.185 | .853 |
|  | Fibrinogen | 1.619* | (0.657) | 2.465 | .014 |
|  | Fibrinogen × Social Support | -0.063** | (0.020) | -3.183 | .001 |
|  | Fibrinogen × Social Strain | 0.056*** | (0.015) | 3.690 | < .001 |
| (Path C) IL-6 Direct Effects | |  |  |  |  |
|  | IL-6 × Social Support | -0.005 | (0.012) | -0.427 | .669 |
|  | IL-6 × Social Strain | 0.010 | (0.009) | 1.078 | .281 |

*Note.* **p* < .05, ****p* < .001.

W1 = wave 1; MSD = Meso Scale Discovery assay; IL-6 = interleukin-6; W2 = wave 2; MDD = major depressive disorder; CRP = C-reactive protein.

# Table S7B

## Total and Mediation Effects of W1 MSD IL-6 Moderation on Social Support and Strain Pathways Predicting W2 MDD Symptoms in Treatment-Adjusted Models

|  | |  | |  | |  | 95% Confidence Interval | |
| --- | --- | --- | --- | --- | --- | --- | --- | --- |
| Pathway | | *b* | % Total | | *p* | | Lower | Upper |
| IL-6 × Social Support | |  |  | |  | |  |  |
|  | Total Effects | -0.015 | 100% | | .215 | |  |  |
|  | Direct Effects | -0.005 | 36.6% | | .669 | |  |  |
|  | Mediation  (CRP + Fibrinogen)^a^ | -0.009 | 63.4% | | Not significant | | -0.037 | 0.003 |
|  | CRP only^b^ | -0.002 |  | |  | |  |  |
|  | Fibrinogen only^b^ | -0.012 |  | |  | |  |  |
| IL-6 × Social Strain | |  |  | |  | |  |  |
|  | Total Effects | 0.020* | 100% | | .026 | |  |  |
|  | Direct Effects | 0.010 | 51.1% | | .281 | |  |  |
|  | Mediation  (CRP + Fibrinogen)^a^ | 0.010^†^ | 48.9% | | Significant | | 0.001 | 0.027 |
|  | CRP only^b^ | 0.004 |  | |  | |  |  |
|  | Fibrinogen only^b^ | 0.010 |  | |  | |  |  |

*Note.* **p* < .05,  ^†^Significant direct effect: 95% CI intervals excluding zero indicate significance.

W1 = wave 1; MSD = Meso Scale Discovery assay; IL-6 = interleukin-6; W2 = wave 2; MDD = major depressive disorder; CRP = C-reactive protein.

^a^Bootstrapped confidence intervals.

^b^Component-wise mediation analyses.

# Table S7C

## Model Comparison of Baseline Moderation and Full Mediation of MSD IL-6 on Social Support and Strain Pathways Predicting W2 MDD Symptoms in Treatment-Adjusted Models

| Model | *Adj. R^2^* | *AIC* | *df* | *SS* | *F* | *p* |
| --- | --- | --- | --- | --- | --- | --- |
| 1: Baseline | 0.150 | 3971.6 |  |  |  |  |
| 2: Full Mediation | 0.170 | 3951.8 | 6 | 77.92 | 5.30*** | < .001 |

*Note*. ****p* < .001.

W1 = wave 1; MSD = Meso Scale Discovery assay; IL-6 = interleukin-6; W2 = wave 2; MDD = major depressive disorder; *Adj. R^2^* = adjusted *R*-squared; AIC = Akaike information criterion; *F* = *F*-ratio statistic.

Model 1 includes the predictors: W1 MDD symptoms, social support, social strain, IL-6 interaction terms, and treatment visit terms. Model 2 adds CRP, fibrinogen, and their interactions with social support and social strain.

# Table S8

## Covariate-Adjusted Models of W1 ELISA IL-6 Levels, MDD Symptoms, Social Support, and Strain with All Relevant Covariates on W2 ELISA IL-6 Levels

| **Linear model estimates** | | | | | |
| --- | --- | --- | --- | --- | --- |
| Parametric coefficients | *b* | *(SE)* | *t* | *p* | *d* |
| (Intercept) | 0.621*** | (0.135) | 4.600 | < .001 | 0.285 |
| W1 MDD Symptoms | 0.002 | (0.008) | 0.211 | .833 | 0.013 |
| W1 Social support | 0.004 | (0.003) | 1.396 | .163 | 0.087 |
| W1 Social strain | -0.003 | (0.003) | -1.308 | .191 | -0.081 |
| W1 IL-6 | 0.168 | (0.107) | 1.569 | .117 | 0.097 |
| Medical treatment visits | 0.008* | (0.004) | 2.166 | .031 | 0.134 |
| Mental health treatment visits | -0.001 | (0.002) | -0.511 | .610 | -0.032 |
| Body mass index | 0.013*** | (0.003) | 5.049 | < .001 | 0.313 |
| History of smoking | 0.091** | (0.029) | 3.160 | .002 | 0.196 |
| Number of chronic conditions | 0.004 | (0.007) | 0.593 | .553 | 0.037 |
| Medication use | 0.030** | (0.011) | 2.619 | .009 | 0.162 |
| W1 Social support × IL-6 | 0.003 | (0.003) | 0.979 | .328 | 0.061 |
| W1 Social strain × IL-6 | -0.000 | (0.003) | -0.188 | .851 | -0.012 |
| *R^2^* | 0.245 |  |  |  |  |
| Adjusted *R^2^* | 0.236 |  |  |  |  |
| *F*-statistic | 28.14*** |  |  |  |  |
| *p* | < .001 |  |  |  |  |
| **Generalized additive model (GAM) non-linear estimates** | | | | | |
| Parametric coefficients | *b* | *(SE)* | *t* | *p* | *d* |
| (Intercept) | 0.863*** | (0.202) | 4.278 | < .001 | 0.265 |
| W1 MDD Symptoms | 0.001 | (0.008) | 0.172 | .864 | 0.011 |
| W1 Social support | 0.003 | (0.003) | 0.925 | .355 | 0.057 |
| W1 Social strain | -0.004 | (0.003) | -1.481 | .139 | -0.092 |
| W1 IL-6 | 0.053 | (0.120) | 0.442 | .659 | 0.027 |
| Medical treatment visits | 0.008* | (0.004) | 2.262 | .024 | 0.140 |
| Mental health treatment visits | -0.001 | (0.002) | -0.327 | .744 | -0.020 |
| Body mass index | 0.014*** | (0.003) | 5.205 | < .001 | 0.323 |
| History of smoking | 0.091** | (0.028) | 3.201 | .001 | 0.198 |
| Number of chronic conditions | 0.002 | (0.007) | 0.283 | .777 | 0.018 |
| Medication use | 0.031** | (0.011) | 2.796 | .005 | 0.173 |
| Significance of smooth terms | *edf* | *rdf* | *F* | *p* |  |
| s(W1 Social support × IL-6) | 4.131 | 4.889 | 3.830** | .003 |  |
| s(W1 Social strain × IL-6) | 1.333 | 1.561 | 0.112 | .858 |  |
| Rank | 29/29 |  |  |  |  |
| Adjusted *R^2^* | 0.250 |  |  |  |  |
| Deviance explained | 0.261 |  |  |  |  |

*Note*. **p* < .05, ***p* < .01, ****p* < .001.

W1 = wave 1; ELISA = enzyme-linked immunosorbent assay; IL-6 = interleukin-6; MDD = major depressive disorder; W2 = wave 2; *edf* = estimated degrees of freedom; *rdf* = reference degrees of freedom; *F* = *F*-ratio statistic.

s() = smoothed terms to accommodate any nonlinear relations.

# Table S9

## Covariate-Adjusted Models of W1 MSD IL-6 Levels, MDD Symptoms, Social Support, and Strain with All Relevant Covariates on W2 MSD IL-6 Levels

| **Linear model estimates** | | | | | |
| --- | --- | --- | --- | --- | --- |
| Parametric coefficients | *b* | *(SE)* | *t* | *p* | *d* |
| (Intercept) | 0.065 | (0.118) | 0.546 | .585 | 0.034 |
| W1 MDD Symptoms | 0.004 | (0.009) | 0.442 | .658 | 0.027 |
| W1 Social support | 0.005 | (0.002) | 1.845 | .065 | 0.114 |
| W1 Social strain | -0.001 | (0.002) | -0.378 | .705 | -0.023 |
| W1 IL-6 | 0.189 | (0.131) | 1.441 | .150 | 0.089 |
| Medical treatment visits | 0.006 | (0.004) | 1.534 | .125 | 0.095 |
| Mental health treatment visits | -0.004* | (0.002) | -1.985 | .047 | -0.123 |
| Body mass index | 0.016*** | (0.003) | 5.717 | < .001 | 0.354 |
| History of smoking | 0.057 | (0.031) | 1.811 | .070 | 0.112 |
| Number of chronic conditions | 0.002 | (0.008) | 0.278 | .781 | 0.017 |
| Medication use | 0.047*** | (0.012) | 3.786 | < .001 | 0.235 |
| W1 Social support × IL-6 | 0.004 | (0.004) | 1.054 | .292 | 0.065 |
| W1 Social strain × IL-6 | -0.004 | (0.003) | -1.588 | .113 | -0.098 |
| *R^2^* | 0.151 |  |  |  |  |
| Adjusted *R^2^* | 0.141 |  |  |  |  |
| *F*-statistic | 15.38*** |  |  |  |  |
| *p* | < .001 |  |  |  |  |
| **Generalized additive model (GAM) non-linear estimates** | | | | | |
| Parametric coefficients | *b* | *(SE)* | *t* | *p* | *d* |
| (Intercept) | 0.134 | (0.218) | 0.615 | .539 | 0.038 |
| W1 MDD Symptoms | 0.001 | (0.009) | 0.144 | .885 | 0.009 |
| W1 Social support | 0.005 | (0.004) | 1.507 | .132 | 0.093 |
| W1 Social strain | -0.004 | (0.003) | -1.322 | .186 | -0.082 |
| Medical treatment visits | 0.006 | (0.004) | 1.533 | .126 | 0.095 |
| Mental health treatment visits | -0.003 | (0.002) | -1.424 | .155 | -0.088 |
| Body mass index | 0.014*** | (0.003) | 4.947 | < .001 | 0.307 |
| History of smoking | 0.049 | (0.031) | 1.604 | .109 | 0.099 |
| Number of chronic conditions | -0.001 | (0.008) | -0.101 | .920 | -0.006 |
| Medication use | 0.046*** | (0.012) | 3.769 | < .001 | 0.234 |
| W1 IL-6 | 0.131 | (0.135) | 0.975 | .330 | 0.060 |
| Significance of smooth terms | *edf* | *rdf* | *F* | *p* |  |
| s(W1 Social support × IL-6) | 5.518 | 6.517 | 5.379*** | < .001 |  |
| s(W1 Social strain × IL-6) | 1.001 | 1.002 | 0.631 | .427 |  |
| Rank | 29/29 |  |  |  |  |
| Adjusted *R^2^* | 0.183 |  |  |  |  |
| Deviance explained | 0.196 |  |  |  |  |

*Note*. ****p* < .001.

W1 = wave 1; MSD = Meso Scale Discovery assay; IL-6 = interleukin-6; MDD = major depressive disorder; W2 = wave 2; *edf* = estimated degrees of freedom; *rdf* = reference degrees of freedom; *F* = *F*-ratio statistic.

s() = smoothed terms to accommodate any nonlinear relations.

**Figure S1**

*Contour and Perspective (3D) Plots of the Interactions between W1 Social Support, Social Strain, and ELISA IL-6 in predicting W2 MDD Symptoms*


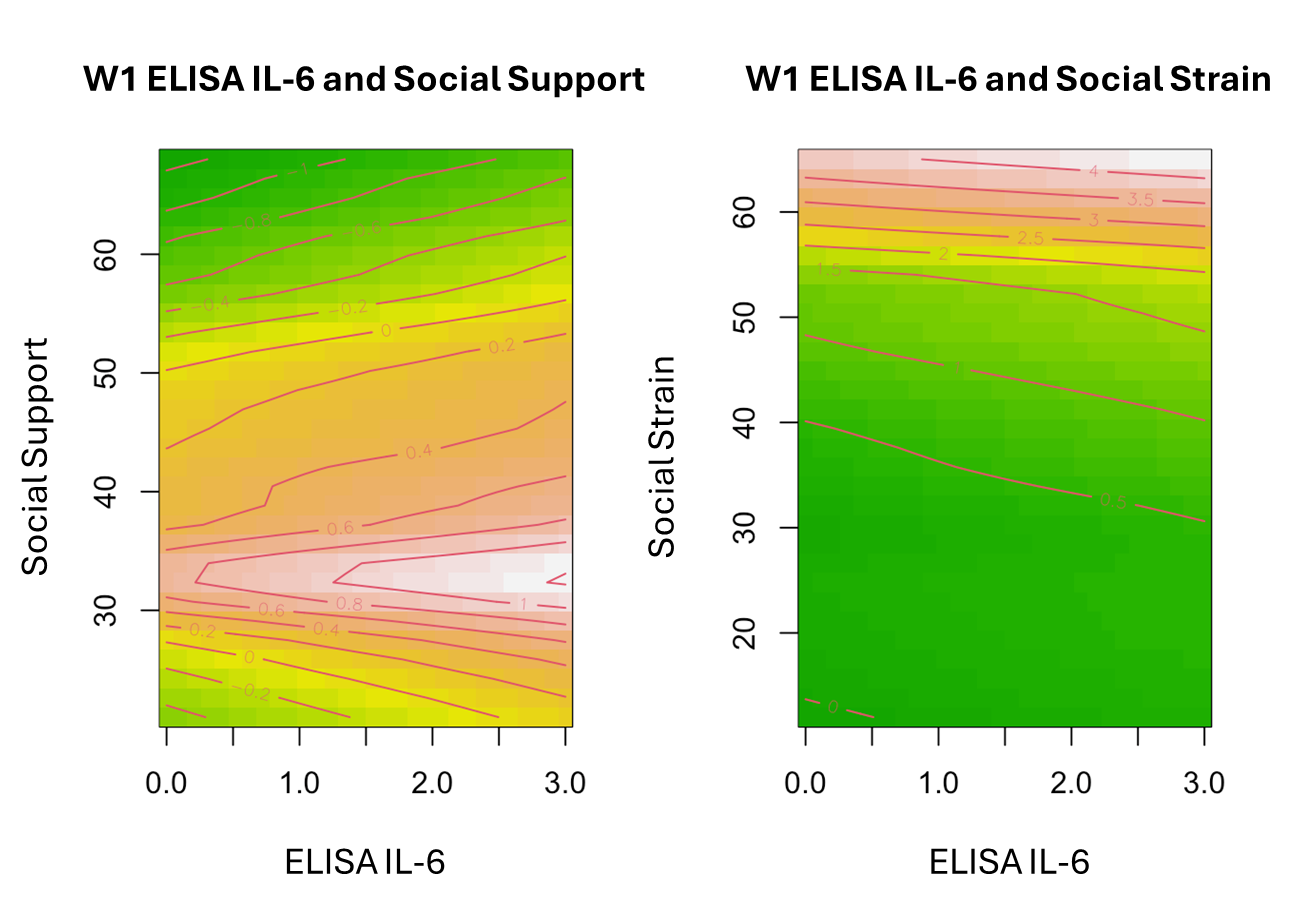


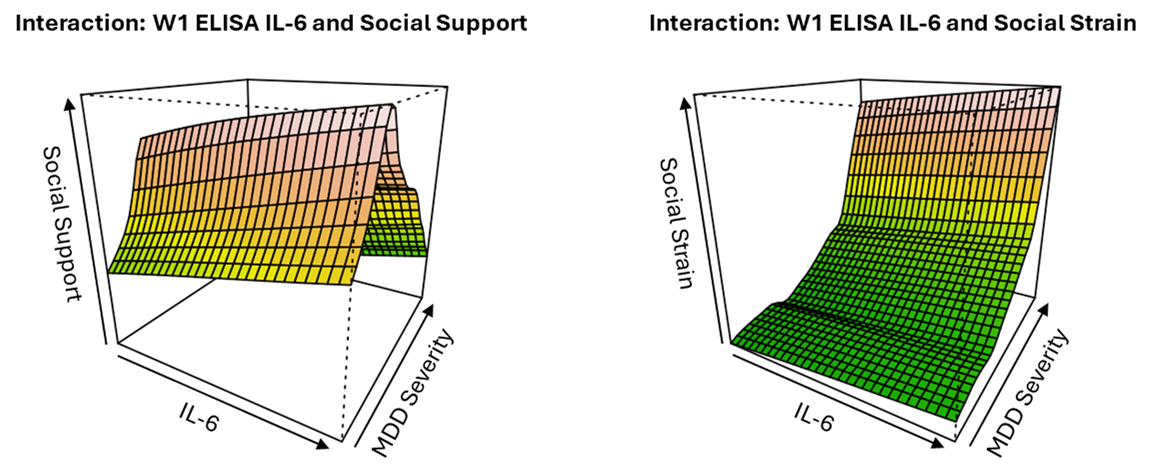


*Note*. ELISA = enzyme-linked immunosorbent assay, IL-6 = interleukin-6, MDD = Major depressive disorder. The color gradient (ranging from green to white) represents the predicted values of MDD severity as a function of both predictors.

**Figure S2**

*Contour and Perspective (3D) Plots of the Interaction between W1 Social Strain and MSD IL-6 in predicting W2 MDD Symptoms*


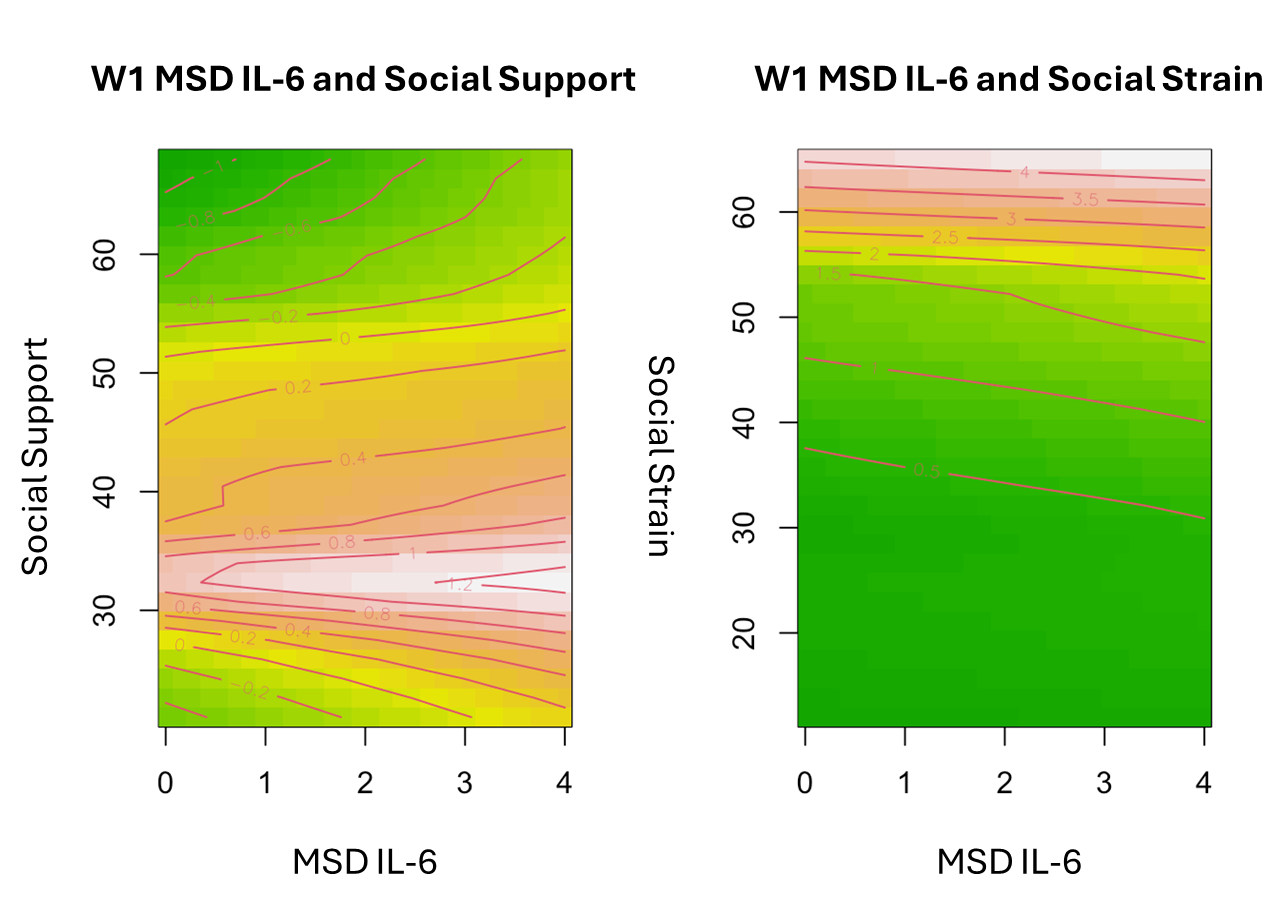


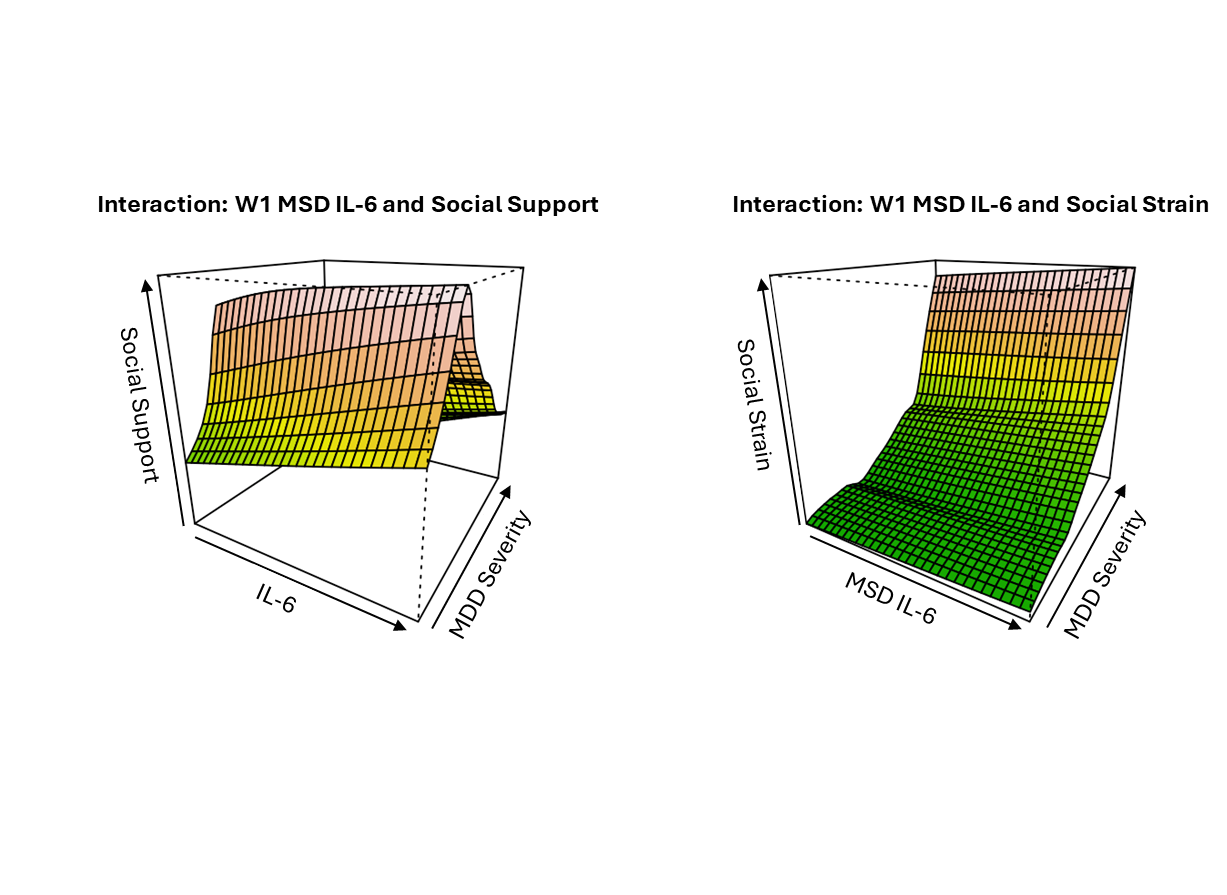


*Note*. MSD = Meso Scale Discovery immunoassay, IL-6 = interleukin-6, MDD = Major depressive disorder. The color gradient (ranging from green to white) represents the predicted values of MDD severity as a function of both predictors.
